# An indolent course of undifferentiated pleomorphic cardiac sarcoma mimicking myxoma: A case report

**DOI:** 10.1016/j.radcr.2026.02.023

**Published:** 2026-03-19

**Authors:** Hengameh Ziaei, Emma Palmquist, Henrik Fagman, Åse A Johnsson, Magnus Hansson, Christoffer Vannas, Martin Westerberg, Erika Fagman

**Affiliations:** a Department of Radiology, Sahlgrenska University Hospital, Region Västra Götaland, Gothenburg, Sweden; b Department of Radiology, Institute of Clinical Sciences, Sahlgrenska Academy, University of Gothenburg, Gothenburg, Sweden; c Department of Clinical Pathology, Sahlgrenska University Hospital, Region Västra Götaland, Gothenburg, Sweden; d Department of Laboratory Medicine, Sahlgrenska Center for Cancer Research, Institute of Biomedicine, Sahlgrenska Academy, University of Gothenburg, Gothenburg, Sweden; e Department of Oncology, Sahlgrenska University Hospital, Gothenburg, Sweden; f Department of Cardiothoracic Surgery, Sahlgrenska University Hospital, Gothenburg, Sweden

**Keywords:** Case report, Cardiac undifferentiated pleomorphic sarcoma, Magnetic resonance imaging, Cardiac tumor, High-grade sarcoma

## Abstract

Cardiac undifferentiated pleomorphic sarcoma (UPS) is a rare and highly malignant tumor, commonly arising in the left atrium. It is associated with poor prognosis, nonspecific symptoms, and difficulty in early diagnosis. Here, we report a case of a 71-year-old woman who presented with palpitations, chest pressure, and exertional dyspnea, in whom initial cardiac magnetic resonance imaging revealed only subtle atrial septal thickening, evaluated as nonspecific, whereas follow-up imaging 2 years later showed progressive growth, ultimately interpreted as a cardiac myxoma. Surgical resection was performed, and histopathologic examination revealed a high-grade UPS with immunohistochemistry and molecular profiling supporting the diagnosis, including amplification of MDM2 and PDGFRA. No metastases were detected, and after multidisciplinary discussion a decision was made to abstain from adjuvant therapy. This case highlights the diagnostic challenge posed by cardiac UPS mimicking benign tumors such as myxoma on imaging and the importance of longitudinal imaging review and a high index of suspicion in cases with atypical radiological features of myxoma. It also raises awareness of an unusually indolent behavior of an UPS that otherwise pursues an aggressive course.

## Introduction

Primary cardiac tumors are rare, with an autopsy incidence between 0.001% and 0.03%. Approximately 10% of primary cardiac tumors are malignant, with cardiac sarcomas being the most common malignant type [1,2]. These tumors typically affect patients between 30 and 50 years of age and are associated with nonspecific symptoms such as dyspnea, palpitations, or thromboembolic events. Median survival is poor, ranging from 3 to 12 months, due to aggressive growth and early metastasis [3,4].

Among these malignancies, cardiac undifferentiated pleomorphic sarcoma (UPS) is a high-grade neoplasm characterized by pleomorphic spindle or epithelioid cells lacking clear lineage of differentiation [5]. Although cardiac UPS is relatively rare, several cases have been previously reported in the literature [[6], [7], [8], [9], [10]]. Notably, some of these have mimicked atrial myxomas radiologically and intraoperatively with myxoid and cystic features, particularly when located in the left atrium [8,10].

Here, we present a case of left atrial UPS that was initially diagnosed as a cardiac myxoma based on cardiac magnetic resonance (CMR) imaging, with retrospective imaging evidence of tumor presence 2 years prior to resection. It emphasizes the diagnostic challenges associated with cardiac UPS and raises awareness of the rare possibility of a more indolent biological behavior in this otherwise aggressive tumor type.

## Case report

A 71-year-old woman was admitted to the thoracic surgery department at Sahlgrenska University Hospital in November 2024 due to a suspected tumor in the atrial septal wall. She had been evaluated over a 2-year period for nonspecific symptoms, including palpitation, fatigue, and exertional dyspnea.

Her medical history included atrioventricular nodal re-entry tachycardia, successfully treated with ablation 8 years earlier, and chronic atrial fibrillation managed medically. She also had a history of left-sided breast cancer treated 2 decades prior with mastectomy, axillary lymph node dissection, adjuvant chemotherapy, and endocrine therapy. A pathogenic CHEK2 mutation was later identified, leading to a prophylactic right-sided mastectomy.

**Fig. 1:**
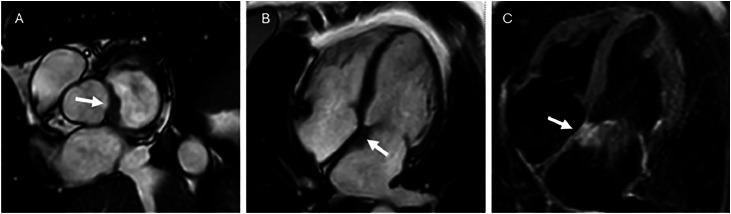
Cardiac magnetic resonance (CMR) showing a subtle thickening of the atrial septum (arrow) in (A) cine steady-state free precession (SSFP) short-axis view and (B) cine SSFP 4-chamber view. (C) Slightly increased signal in short tau inversion recovery (T2 STIR) 4-chamber view.

**Fig. 2:**
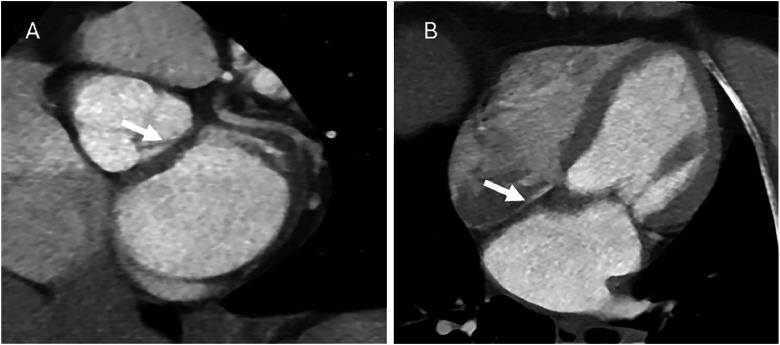
(A and B) Electrocardiography (ECG)-gated cardiac CT revealed slight thickening at the atrial septum (arrow), interpreted as a partial volume effect.

In 2022, she presented with palpitations and fatigue. A transthoracic echocardiogram showed normal-sized ventricles and mild mitral regurgitation, while electrocardiography demonstrated atrial fibrillation with frequent ventricular extrasystoles. An exercise stress test showed no signs of ischemia, arrhythmia, or hemodynamic instability. To further assess for structural pathology, CMR was performed, revealing a small (1.5 × 2 cm^2^) focal thickening in the left atrium near the mitral valve annulus with a slightly elevated T2 signal (Fig. 1). No other pathological findings were detected. A subsequent CT did not confirm a lesion; however, subtle thickening at the atrial septum was noted, which was assumed to be due to partial volume effect (Fig. 2). As no definitive pathology was confirmed, further investigation was not performed at that time.

**Fig. 3:**
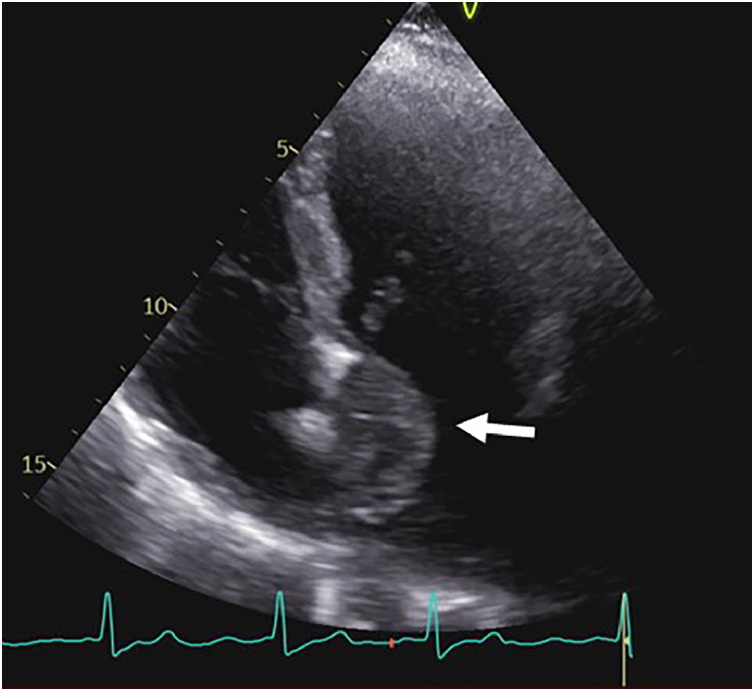
Transthoracic echocardiography (TTE) demonstrating a large, heterogeneous, multilobulated, semimobile mass with attachment to the septal wall of the left atrium and mitral valve annulus in a 4-chamber view (arrow).

**Fig. 4:**
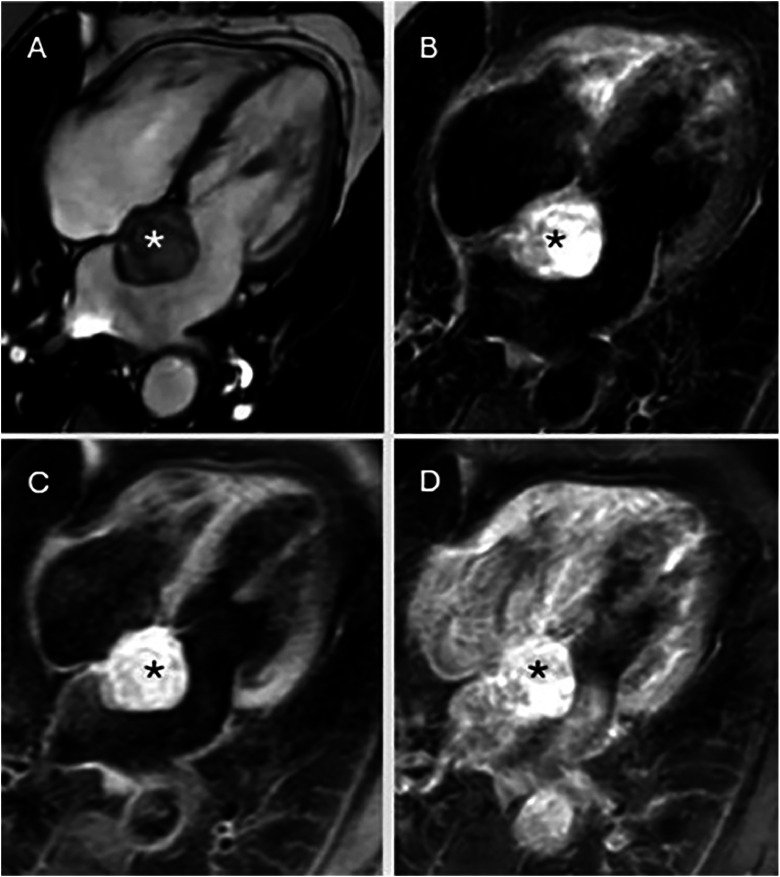
Cardiac magnetic resonance (CMR) images: (A) SSFP 4-chamber view showing the mass within the left atrium (*). (B) T2 STIR, 4-chamber view demonstrating high T2 signal within the tumor, suggesting edema or increased water content. (C) T1-weighted sequence without contrast showing high heterogeneous signal intensity within the tumor. (D) T1-weighted sequence with contrast showing slightly diffuse inhomogeneous gadolinium enhancement.

By the summer of 2024, the patient experienced worsening dyspnea and palpitations. A follow-up transthoracic echocardiogram identified a left atrial mass (Fig. 3). CMR confirmed a 3.5 × 4 cm^2^ mass within the left atrium, attached to the interatrial septum at the level of the foramen ovale, extending anteriorly toward the anterior leaflet of the mitral valve, where it exhibited a broad-based attachment. The lesion was otherwise rounded and demonstrated heterogeneous signal characteristics. It exhibited high signal intensity on both T2- and T1-weighted sequences, along with irregular contrast enhancement. Although the lesion was already hyperintense on precontrast T1-weighted images, enhancement after gadolinium administration was determined by side-by-side comparison of pre- and postcontrast series, demonstrating a further heterogeneous increase in signal intensity within the mass relative to the adjacent myocardium and blood pool. Small areas within the mass lacked contrast enhancement but were markedly hyperintense on T2-weighted images, suggesting cystic or myxoid components (Fig. 4). The suggested radiological diagnosis was cardiac myxoma.

Coronary angiography ruled out significant coronary artery disease, and the patient was accepted for surgery. Intraoperatively, the tumor showed a very broad-based attachment to both the atrial septum and the anterior mitral valve leaflet, which, together with the macroscopic appearance, raised suspicion of malignancy. The majority of the mass attached to the atrial septum was successfully resected. However, a portion of the lesion was found to be strongly adherent to the anterior leaflet of the mitral valve. Radical removal of the mass would have required an extensive surgical procedure, including replacement of the mitral valve and potentially the aortic root. Given the complexity and additional risks, this extended resection was not performed at the time.

**Fig. 5:**
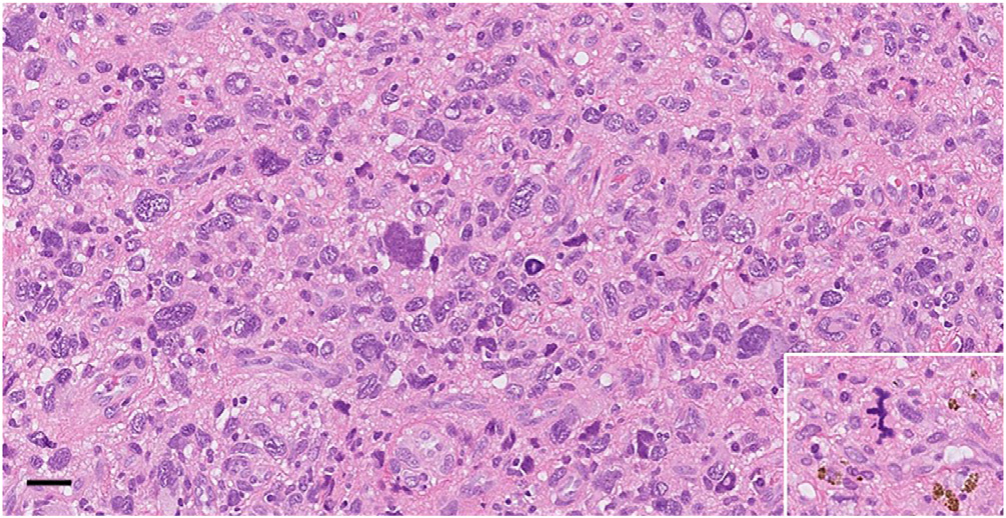
H&E staining showing tumor morphology with pleomorphic cells in a collagenous stroma. Insert shows an atypical mitosis and hemosiderin depositions. Scale bar = 50 µm.

Routine histopathology of the 3.7 × 3.3 × 2.0 cm^3^ resection specimen revealed the morphology of a pleomorphic sarcoma (Fig. 5). The tumor was heterogeneous with fibrin layering, bleeding, and hemosiderin depositions. Vaguely spindle-shaped, atypical cells were admixed with markedly pleomorphic cells with enlarged and hyperchromatic nuclei and abundant infiltrating lymphocytes set in a collagenous stroma. The mitotic activity was moderate (4 mitoses per 10 high-power fields [10 HPF = 1,734 mm²]) with occasional atypical mitoses. No definitive tumor cell necrosis was evident. Immunohistochemical staining was positive for CD163, whereas AE1/3, GATA3, smooth muscle actin, desmin, caldesmon, MYF4, calretinin, and WT1 were all negative. The Ki67 index was 12% in hotspots. Comprehensive genomic profiling by DNA sequencing (GMS560 target capture NGS-panel covering >550 genes) showed abundant numerical and segmental copy number alterations with high-level amplification (35-50 copies) of MDM2 and PDGFRA and lower level amplification (10-15 copies) of KIT, CCND1, GAB2, and YAP1. CDK4 was not amplified. The tumor was microsatellite stable, and the mutational burden was low (0.8 mutations/Mb). The findings were thus collectively consistent with cardiac UPS [11].

## Follow-up and outcome

**Fig. 6:**
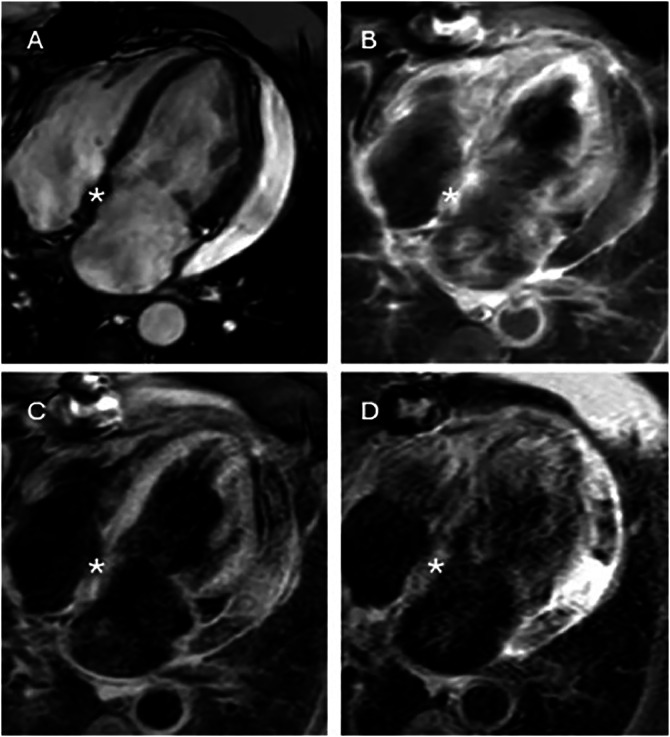
Cardiac magnetic resonance (CMR) images postsurgery: (A) SSFP 4-chamber view shows a remaining thickening of the atrial septum at the previous location of the mass (*). (B) Short tau inversion recovery (T2 STIR) 4-chamber view demonstrating slightly high signal in the same area. (C) T1-weighted sequence without contrast showing heterogeneous diffuse high signal. (D) T1-weighted sequence with contrast slightly diffuse inhomogeneous gadolinium enhancement. Note that there is also pericardial effusion.

Postoperatively, the patient developed pleural and pericardial effusion, which gradually resolved. ^18^F-FDG PET/CT showed no evidence of distant metastases. At the same time, a follow-up CMR indicated contrast uptake in the atrial septal wall. However, it was not evident whether this represented a remaining tumor or postsurgical alterations (Fig. 6). The patient was referred to the oncological department for consideration of adjuvant therapy.

Given the aggressive nature of UPS and the limited evidence for adjuvant therapy, the case was reviewed by a national tumor board. The initial recommendation was adjuvant radiotherapy to the heart (1.8 Gy to a total of 45 Gy), followed by chemotherapy. However, after further discussions, a decision was made to abstain from adjuvant therapy due to a lack of strong evidence and the patient’s perspective. The team recommended follow-up radiological evaluations every 2-3 months to monitor for potential recurrence.

In the 6 months after surgery, 3 follow-up CMRs have been conducted without any obvious signs of local recurrence. Follow-up CT scans of the chest, abdomen, and brain have not shown any suspicion of metastatic disease.

## Discussion

Primary cardiac tumors are rare, with only about 10% being malignant, most of which are sarcomas [12]. Among them, UPS is a highly aggressive mesenchymal neoplasm that predominantly arises in the left atrium, particularly on the posterior or lateral walls [10]. These tumors often manifest in the sixth decade of life and present with nonspecific symptoms such as dyspnea, palpitations, syncope, and signs of heart failure, as well as complications, including arrhythmias, obstruction, and distant metastases [13]. Given their rarity and nonspecific clinical presentation, diagnosis is frequently delayed, and clinical management is challenging.

The initial diagnostic evaluation typically relies on multimodality imaging, including echocardiography, CMR, and CT [1]. CMR offers superior soft-tissue characterization and is central in differentiating benign from malignant cardiac lesions [3,[14], [15], [16]]. Cardiac sarcomas typically appear isointense to the myocardium on T1-weighted images. However, areas of intramural hemorrhage or necrosis contribute to a heterogeneous appearance. In our case, the lesion demonstrated relatively high signal intensity on precontrast T1-weighted imaging, which we presume reflects intratumoral hemorrhage and blood degradation products, consistent with the histopathological findings of fibrin layering, bleeding, and hemosiderin depositions within the tumor. On T2 images, sarcomas exhibit high signal intensity due to edema, necrosis, or myxoid components [14]. Nevertheless, these imaging features are not pathognomonic, as cardiac myxomas, the most common benign cardiac tumor, can also exhibit heterogeneous signal due to variable internal composition [16,17]. This overlap is particularly problematic because cardiac UPS, like myxoma, most commonly occurs in the left atrium [10] thus increasing the risk of diagnostic confusion.

In this case, the tumor was initially presumed to be a cardiac myxoma based on its radiological features on CMR in 2024. Retrospective analysis of prior imaging from 2022 revealed a subtle but distinct lesion in the same place. The slow growth rate further supported the initial working diagnosis of myxoma, even though the appearance at the initial examination was unusual for a myxoma, with a diffuse thickening of the atrial wall instead of the expected appearance, which is a pedunculated lobulated lesion. Definitive diagnosis was ultimately achieved through histopathologic examination, which revealed high-grade UPS.

From a pathogenetic standpoint, cardiac UPS is thought to arise through complex genomic alterations rather than along a single defined molecular pathway. In our case, molecular profiling demonstrated high-level amplification of MDM2 and PDGFRA, consistent with dysregulation of cell-cycle control and growth factor signaling. Notably, although the patient had a history of left-sided breast cancer treated 2 decades earlier with mastectomy, axillary lymph node dissection, adjuvant chemotherapy, and endocrine therapy, she did not receive radiotherapy. This is relevant because radiation therapy is a well-known risk factor for secondary sarcomas, including rare cardiac sarcomas, typically arising within the prior radiation field after a latency of up to 2 decades. As no radiation was given to the patient, the present case thus represents a de novo cardiac UPS.

This case underscores the potential for UPS to mimic atrial myxomas, both radiologically and clinically. Several case reports have described similar diagnostic challenges [[8], [9], [10]]. For instance, Kerbl et al. [8] reported a left atrial lesion with imaging features in favor of atrial myxoma, ultimately diagnosed as UPS after histopathological examination. Similarly, Vallés-Torres et al. [10] described such a tumor mistaken for atrial myxoma due to benign imaging characteristics and indolent onset. A summary of the clinical presentation and key imaging features of these reports is provided in Table 1. These reports highlight the radiologic overlap between benign and malignant cardiac tumors and emphasize the need for combining imaging, clinical context, and histopathology in ambiguous cases. In uncertain cases, advanced imaging modalities such as ^18^F-FDG PET/CT or ^18^F-FDG PET-CMR may provide valuable information, as malignant tumors often demonstrate increased metabolic uptake, whereas myxomas typically exhibit minimal or no uptake [18].

**Table 1:** Reported cases of cardiac undifferentiated pleomorphic sarcoma initially misinterpreted as other diagnoses.

Reference	Age/Sex	Location	Clinical presentation	Imaging	Key findings
Kerbl et al. [8]	50-year-old male	LA	Progressive chest pain, dyspnea	TTE, TEE, MRI	2.3 × 2.5 cm^2^ heterogeneous mass on echocardiography; MRI showed no signs of invasion or early enhancement, suggestive of myxoma
Watson et al. [9]	61-year-old female	LA	Progressive dyspnea, lower extremity edema	TTE, CT	Large amorphous filling defect on CT initially presumed thrombus; 3.7 × 3.2 cm^2^ mass causing severe mitral dysfunction
Vallés-Torres et al. [10]	60-year-old female	LA	Progressive dyspnea, chest discomfort	TTE, TEE, MRI	8.0 × 4.5 × 4 cm^3^ mobile mass on echocardiography; MRI showed homogeneous enhancement, smooth margins, and no signs of invasion, suggestive of myxoma

CT, computed tomography; LA, left atrium; MRI, magnetic resonance imaging; TTE, transthoracic echocardiography; TEE, transesophageal echocardiography.

An unusual feature in our case was the absence of metastatic disease, despite the tumor being radiologically detectable for approximately 2 years prior to surgical intervention. This indolent course stands in sharp contrast to the typical clinical behavior of cardiac UPS, which is generally characterized by aggressive growth, early metastasis, and poor prognosis [1,13].

Nonetheless, both the histopathological features and the genetic profile in the present case were consistent with a diagnosis of UPS, thereby confirming its classification despite the atypical clinical behavior. Interestingly, emerging evidence suggests that a subset of UPS may exhibit a more indolent biological behavior, particularly following treatment. For example, Nupur et al. [19] reported a case of cardiac UPS with no recurrence 40 months after complete surgical resection followed by adjuvant chemotherapy and radiotherapy. Similarly, Vaitiekienė et al. [20] described a patient with primary cardiac sarcoma who remained disease-free for over 26 months posttreatment. Taken together, these cases, including this one, suggest that not all cardiac UPS follow a dismal clinical course, which raises the possible existence of a biologically indolent UPS subtype. Recognition of this potential variability is clinically significant, as it may impact prognostic stratification and inform tailored therapeutic decision-making.

In conclusion, although rare, cardiac sarcoma should always be considered in the differential diagnosis of atrial masses, particularly when imaging characteristics diverge from the typical features of myxoma. Our case adds to the growing body of literature suggesting that not all cardiac UPS follow a dismal course and underscores the importance of early recognition, comprehensive imaging assessment, and the integration of histopathological and molecular analyses to guide timely and appropriate management.

## Author contributions

All authors played a significant role in the conception, design, analysis, and interpretation of this case report and have approved the final version of the manuscript.

## Patient consent

Written informed consent was duly obtained from the patient for using her data and images for publication of this case report. A copy of the written consent form is available for review by the editor-in-chief of this journal upon request.
